# Factors influencing patient and health care delays in Oropharyngeal Cancer

**DOI:** 10.1186/s40463-020-00413-w

**Published:** 2020-04-23

**Authors:** Markus Nieminen, Timo Atula, Leif Bäck, Antti Mäkitie, Lauri Jouhi, Katri Aro

**Affiliations:** 1 Department of Otorhinolaryngology – Head and Neck Surgery, University of Helsinki and Helsinki University Hospital, PO Box 263, FI-00029 HUS Helsinki, Finland; 2 Division of Ear, Nose, and Throat Diseases, Department of Clinical Sciences, Intervention, and Technology, Karolinska Institutet, Karolinska University Hospital, Stockholm, Sweden; 3 Research Program in Systems Oncology, Faculty of Medicine, University of Helsinki, Helsinki, Finland

**Keywords:** Patient delay, Treatment delay, Primary health care, Diagnostic delay, Oropharyngeal cancer, Human papillomavirus

## Abstract

**Background:**

The incidence of human papillomavirus (HPV)–associated oropharyngeal squamous cell carcinoma (OPSCC) is increasing. Patients with HPV-associated and HPV-unassociated OPSCC differ in many aspects, which may also impact their diagnostic and management timelines. This study aims at studying the patient, primary health care (PHC) and specialist-care (SC) delays and possible differences between these two patient groups in seeking medical care.

**Methods:**

We reviewed all new patients with OPSCC treated between 2016 and 2018 at our institute, which covers a referral area of 1.6 million people. We collected data on patients’ symptoms and factors influencing why they sought medical care using a patient-reported questionnaire and hospital records. We compared delays based on patient and tumor characteristics.

**Results:**

In our study population of 83 patients, the median patient delay was 30 days (range, 0–366), with a median PHC delay of 15 days (range, 0 days–2.5 years), and a median SC delay of 54 days (range, 12–231). The SC delay was further divided into diagnostic hospital delay and treatment delay, each with a median length of 16 days (range, 0–237) and 29 days (range, 0–73), respectively. Furthermore, we found that p16 status did not associate with delays. A longer patient delay associated with specific tumor factors, such as a larger primary tumor and a lower UICC 7th edition stage. Patients that had multiple visits or did not have a follow-up visit scheduled at the initial appointment had longer PHC delays. Treatment delay was significantly longer for patients scheduled for (chemo-)radiotherapy than for those undergoing surgery with or without (chemo-)radiotherapy.

**Conclusions:**

Although delays remained short for the majority of OPSCC patients, long delays require further evaluation and improvement of management. Awareness of presenting symptoms among cancer risk patients and prompt referral practice or a follow-up visit at PHC represent key factors to shortening these delays. Ultimately, the causes for delays in SC appear multifactorial and require institutional quality control.

## Introduction

The incidence of newly diagnosed oropharyngeal squamous cell carcinoma (OPSCC) has increased in Western societies due to the increase in the incidence of high-risk human papillomavirus (HPV)–associated OPSCC [1–5]. Traditionally, OPSCC has been associated with smoking and the heavy use of alcohol, especially the HPV-unassociated OPSCC. Patients with HPV-associated OPSCC, however, are typically younger, smoke less [6, 7], present with smaller primary tumors with more advanced regional lymph node spread [7–9], and have up to a 50% better survival rate than patients with HPV-unassociated OPSCC [10]. Therefore, these two subgroups may represent two distinct disease entities [11].

A marked delay before the initiation of curative treatment among head and neck cancer (HNC) patients may lead to tumor growth, disease advancement, and impaired prognosis [12–20]. Treatment delay falls into two categories: patient- and health care–related delays [21]. Health care–related delays can be further divided into primary health care (PHC) delay and specialist-care (SC) delay. According to a meta-analysis by Stefanuto et al. [16], patient delay is the most important factor causing delay before treatment, a finding similar to our previous study on delays including all HNC sites [22]. Since patient and tumor characteristics differ markedly within the two OPSCC subgroups, it seems reasonable to assume that these patients seek medical care in different ways.

This study focuses on OPSCC patients’ total delay before treatment, from the onset of initial symptoms to the initiation of curative treatment. We were specifically interested in determining how existing delays related to patient or tumor characteristics. Therefore, we explored whether any differences between patient groups with HPV-associated and HPV-unassociated OPSCC existed.

## Patients and methods

We identified a total of 111 patients with newly diagnosed OPSCC seen at the Department of Otorhinolaryngology – Head and Neck Surgery, Helsinki University Hospital (Helsinki, Finland) between 14 January 2016 and 14 January 2018. We did not include patients with a previous HNC (*n* = 2) or incapable of understanding or completing the questionnaire due to dementia (*n* = 4), a language barrier (*n* = 3) or other reasons (*n* = 2). Another 17 patients did not return the questionnaire. The remaining 83 patients formed our study cohort, the characteristics of whom appear in Tables 1 and 2. Patient and PHC delays were analyzed among all patients regardless of treatment intent. SC delay was analyzed only among patients with a curative treatment intent.

**Table 1:** Patient characteristics according to p16 status (*n* = 81 patients)

	p16-positive, *n* = 64 (%)	p16-negative, *n* = 17 (%)	*P* value	All, *n* = 83 (%)
Sex				
Male	46 (71.9)	12 (70.6)	1.000	60 (72.3)
Female	18 (28.1)	5 (29.4)		23 (27.7)
Mean age (in years)	59.7	66.6	**0.023**	61.4
History of smoking				
Never smoked	23 (35.9)	2 (11.8)	**< 0.001**	26 (31.3)
Former smoker	33 (51.6)	5 (29.4)		39 (47.0)
Current smoker	8 (12.5)	10 (58.8)		18 (21.7)
Excessive use of alcohol				
No	46 (71.9)	4 (23.5)	**< 0.001**	52 (62.6)
Yes	11 (17.2)	6 (35.3)		17 (20.5)
Previous use	7 (10.9)	7 (41.2)		14 (16.9)
T class^b^				
T1–2	47 (73.4)	13 (76.5)	1.000	61 (73.5)
T3–4	17 (26.6)	4 (23.5)		22 (26.5)
N class^b^				
N+	10 (15.6)	6 (35.3)	0.090	16 (19.3)
N0	54 (84.4)	11 (64.7)		67 (80.7)
Stage^b^				
I-II	6 (9.4)	5 (29.4)	**0.047**	11 (13.3)
III-IV	58 (90.6)	12 (70.6)		72 (86.7)
Histological grade^c^				
I	1 (1.6)	2 (12.5)	**0.005**	3 (3.7)
II	9 (14.3)	6 (37.5)		16 (19.8)
III	53 (84.1)	8 (50.0)		62 (76.5)

^a^In 2 patients, p16 staining was unavailable

^b^According to UICC, 7th edition

^c^*n* = 81; p16-positive (*n* = 63), p16-negative (*n* = 16)

**Table 2:** Delay (in days) and patient characteristics (*n* = 83 patients)

	Number (%)	Patient delay	*P* value	PHC^a^ delay	*P* value	Specialist-care delay^b^	*P* value
Age (in years)			0.370		0.974		0.253
< 46	9 (10.8)	30.0		10.0		80.0	
46–65	38 (45.8)	24.0		15.0		54.5	
> 65	36 (43.4)	31.0		16.0		50.0	
Sex			0.795		0.305		0.806
Male	60 (72.3)	30.0		15.0		54.0	
Female	23 (27.7)	26.5		21.0		50.0	
History of smoking			0.327		0.799		0.069
Never smoked	26 (31.3)	30.0		13.0		62.0	
Former smoker	39 (47.0)	20.0		16.0		51.0	
Current smoker	18 (21.7)	39.5		15.5		49.0	
Excessive use of alcohol			0.498		0.927		0.608
No	52 (62.7)	23.0		18.0		54.5	
Yes	17 (20.5)	34.0		15.0		52.5	
Previous use	14 (16.9)	38.5		12.5		48.0	
Education			0.773		0.987		0.500
Primary school	27 (32.5)	30.5		16.0		59.0	
Secondary education^c^	36 (43.4)	31.0		14.0		49.0	
Post-secondary education^d^	20 (24.1)	24.0		20.0		52.5	
Employment			**0.043**		0.414		0.866
Currently employed or studying	29 (34.9)	20.0		14.5		55.0	
Unemployed or retired	53 (63.9)	34.0		17.0		52.5	
Unknown	1 (1.2)						
Place of residence			0.342		0.311		0.759
Capital area	50 (60.2)	30.0		16.0		50.0	
Other	33 (39.8)	28.0		14.0		62.0	

^a^*PHC* primary health care

^b^*n* = 77 patients

^c^Senior high school

^d^University or university of applied sciences

Data were collected from questionnaires and from hospital medical records. Patients received a questionnaire before the initiation of definite treatment. If the patient did not return the questionnaire within a reasonable time, a reminder was sent via mail. Fixed-choice questions were posed on symptoms, the time of the appearance of symptoms or signs, the time of the initial visit to a physician (or dentist, hospital emergency unit or private otorhinolaryngologist), the number of visits to a physician before referral to the Department of Otorhinolaryngology – Head and Neck Surgery (Dept. ORL-HNS), the patient’s education level, and employment. The Research Ethics Board at the Hospital District of Helsinki and Uusimaa approved the study design (record number: 398/13/03/02/15) and an institutional permit was granted to complete this study. All participants in this study signed a written consent form.

The data collected from medical records included patient-related variables such as age, sex, history of smoking, the use of alcohol, and place of residence (Table 2). Tumor-related variables included the tumor site (documented using ICD-10 classification codes), the diameter and invasion to surrounding tissues (T class), the presence of regional and distant metastases (N and M classes), stage, and p16 immunohistochemical staining status (Table 3). During our data collection period, UICC released the 8th edition of the TNM classification; therefore, variables for T, N, and M classes were assessed using both the 7th and 8th editions [11, 23]. In this study, we used p16 immunohistochemistry to divide patients into p16-positive and p16-negative groups [24]. The tumor site was divided into subsites as follows: lateral wall (palatine tonsils and tonsillar pillars), anterior wall (base of the tongue and vallecula), superior wall (soft palate and uvula), and posterior wall. The most common tumor site was the lateral wall of the oropharynx (*n* = 47; 57%). The majority (*n* = 64; 77%) of tumors were p16-positive. Table 3 summarizes the other tumor-related characteristics.

**Table 3:** Delay (in days) and tumor characteristics (*n* = 83 patients)

	Number (%)	Patient delay	*P* value	PHC^a^ delay	*P* value	Specialist-care delay^b^	*P* value
Site			0.216		0.130		0.632
Anterior wall	32 (38.6)	31.0		27.0		48.5	
Lateral wall	47 (56.6)	16.5		13.5		55.0	
Posterior wall	1 (1.2)						
Superior wall	0						
Overlapping sites (C10.8)	3 (3.6)	76.0		10.0		58.0	
T Class, 7th and 8th UICC editions^c^			**0.031**		0.963		0.676
T1-T2	61 (73.5)	22.5		15.5		54.5	
T3-T4	22 (26.5)	59.0		15.0		50.0	
N class, 7th UICC edition			0.064		0.587		0.752
N0	16 (19.3)	48.0		15.0		54.0	
N1	12 (14.5)	15.0		14.0		50.0	
N2a	6 (7.2)	39.0		18.0		51.5	
N2b	30 (36.1)	7.0		16.0		54.5	
N2c	18 (21.7)	47.0		15.0		54.0	
N3	1 (1.2)						
N class, 8th UICC edition			**0.020**		0.746		0.184
N0	16 (19.3)	48.0		15.0		54.0	
N1	41 (49.4)	15.5		15.5		54.0	
N2	13 (15.7)	38.5		15.0		54.5	
N2a	2 (2.4)	180.5		18.0			
N2b	5 (6.0)	3.0		8.0		50.0	
N2c	5 (6.0)	75.0		15.0		39.0	
N3	1 (1.2)						
M class, 7th and 8th UICC editions^c^			0.083		0.440		
M0	79 (95.2)	28.0		15.0		52.5	
M1	4 (4.8)	75.5		13.0			
Stage, 7th UICC edition			**0.047**		0.939		0.644
I	2 (2.4)	125.5		76.5			
II	9 (10.8)	31.0		14.0		45.0	
III	11 (13.3)	13.0		15.0		59.0	
IV A	54 (65.1)	23.0		17.0		54.0	
IV B	3 (3.6)	59.0		9.0		37.0	
IV C	4 (4.8)	75.5		13.0			
Stage, 8th UICC edition			0.285		0.737		0.285
I	44 (53.0)	19.0		14.5		55.0	
II	9 (10.8)	31.0		21.0		64.0	
III	14 (16.9)	53.0		14.0		48.0	
IV	2 (2.4)	157.0		10.0			
IV A	12 (14.5)	14.0		15.0		49.5	
IV B	0						
IV C	2 (2.4)	75.5		13.0			
p16 status			0.280		0.580		0.160
Positive	64 (77.1)	24.0		15.0		54.0	
Negative	17 (20.5)	37.0		16.0		45.5	
Unknown	2 (2.4)						
Histological grade			0.257		0.424		0.749
I	3 (3.6)	151.0		22.0		59.0	
II	16 (19.3)	28.0		34.0		56.0	
III	62 (74.7)	27.0		14.5		50.0	
Unknown	2 (2.4)						

^a^*PHC* primary health care

^b^*n* = 77 patients

^c^T and M classes are similar according to the UICC 7th and 8th editions

### Definition of delay intervals

**Fig. 1:**
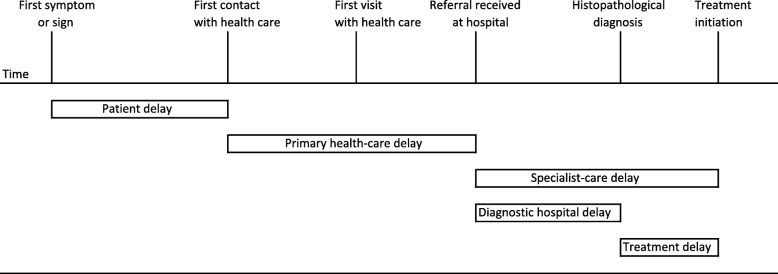
The definitions of patient, primary health care (PHC) and specialist-care (SC) delays used in our study series of OPSCC patients (*n* = 83)

We used the following time intervals: patient delay, or the time interval between the appearance of the first symptom or sign of disease and the initial contact with a health care provider; PHC delay, or the time interval between the initial contact with a health care provider and the referral to the Dept. ORL-HNS; and SC delay, or the time interval between the referral to the Dept. ORL-HNS and the initiation of curative treatment (Fig. 1). We further divided SC delay into the diagnostic hospital delay, or the time interval between the referral to the SC clinic and histopathological diagnosis, and treatment delay, or the time interval between diagnosis and the initiation of curative treatment.

We double-checked the delay data from hospital records in order to minimize recall bias. We noted a discrepancy in a few cases between patient-reported delay times on the questionnaire and the delay times reported at the initial visit to the otorhinolaryngologist. We used the latter for our dataset, since it was documented closer to the onset of symptoms and before the cancer diagnosis.

We used SPSS version 24 (SPSS Inc., Chicago, IL, USA) in the statistical analysis. The distributions of delays skewed to the right (most patients reported shorter delays than average). Therefore, we employed nonparametric tests in our univariate analysis. When analyzing the delay in two independent groups, we employed the Mann–Whitney U test; when analyzing more than two independent groups, we employed the Kruskal–Wallis test. The post-hoc *P* values of the Kruskal–Wallis test were Bonferroni corrected. We reported the patient delays, PHC delays, and SC delays using the median values. A multivariate linear model was employed in the covariate adjusted data analysis. First, we performed a natural log transformation for delay variables. Factors with a *P* < 0.2 in the univariate analysis were included in the multivariate analysis. Specific symptoms were not included in the multivariate analysis since they were considered a result of the disease. The results of the multivariate analysis are reported as adjusted geometric means with 95% confidence intervals. We considered *P* < 0.05 as statistically significant.

## Results

Among the 83 patients in our study cohort, the median patient delay was 30 days (mean, 56; range, 0–366). The univariate analysis revealed that patient-related factors had no impact on the median patient delay (Table 2). In addition, the p16 status did no impact on patient delay. Tumor characteristics that significantly correlated with the patient delay included T class, N class (according to the UICC 8th edition), and stage (according to the UICC 7th edition; Table 3). The most common symptoms included a lump on the neck (*n* = 57; 69%), pain (*n* = 40; 48%), and problems swallowing (*n* = 30; 36%). Table 4 summarizes the symptoms and their relation to a delay. The longer patient delay significantly associated with weight loss and difficulties breathing.

**Table 4:** Delay (in days) and symptoms (*n* = 83 patients)

	Number (%)	Patient delay	*P* value	PHC^a^ delay	*P* value	Specialist-care delay^b^	*P* value
Lump on the neck			0.130		0.764		**0.029**
No	26 (31.3)	31.0		16.0		64.0	
Yes	57 (68.7)	24.0		15.0		49.5	
Pain			0.526		0.898		0.345
No	43 (51.8)	22.5		15.0		55.0	
Yes	40 (48.2)	37.0		16.0		49.5	
Problems swallowing			0.296		0.101		0.268
No	53 (63.9)	24.0		11.0		50.0	
Yes	30 (36.1)	42.0		20.0		60.5	
Hoarseness			0.149		0.683		0.252
No	70 (84.3)	24.0		15.0		50.0	
Yes	13 (15.7)	68.5		18.0		60.5	
Ulcer or other lesion			0.661		0.633		0.886
No	70 (84.3)	29.0		15.0		51.0	
Yes	13 (15.7)	39.0		20.0		55.0	
Weight loss			**0.012**		0.837		0.391
No	74 (89.2)	24.0		15.0		52.5	
Yes	9 (10.8)	75.5		16.0		69.0	
Bleeding			0.959		0.491		0.155
No	79 (95.2)	30.0		15.0		54.0	
Yes	4 (4.8)	28.5		26.0		41.5	
Difficulty breathing			**0.007**		0.075		0.688
No	79 (95.2)	29.0		15.0		51.0	
Yes	4 (4.8)	151.0		35.0		64.0	
Facial swelling			0.583		0.223		
No	81 (97.6)	30.0		15.5		52.5	
Yes	2 (2.4)	51.5		5.0			
Visual impairment			0.627		0.916		
No	82 (98.8)	30.0		15.0		54.0	
Yes	1 (1.2)	9.0		19.0			
Other symptoms	16 (19.3)						
Pain intensity			0.791		0.345		0.561
No pain	42 (50.6)	23.0		15.0		54.5	
Mild or moderate^c^	29 (34.9)	39.5		14.5		49.5	
Severe^d^	12 (14.5)	21.0		34.5		54.0	
Number of different symptoms			**0.021**		0.669		0.652
One	28 (33.7)	14.0		10.0		50.0	
Two	25 (30.1)	30.5		15.5		54.0	
Three or more	30 (36.1)	59.0		17.5		53.5	

^a^*PHC* primary health care

^b^*n* = 77 patients

^c^No need for regular painkillers

^d^Regular painkillers needed

The multivariate analysis revealed that patients with a lower stage or multiple symptoms had a longer adjusted mean patient delay (Table 5). Since T class, N class, and M class also emerged as suitable for the multivariate analysis, we created an alternative model including these variables, but neither dichotomous T class (T3–4 vs T1–2, *P* = 0.431), N class (N+ vs N0, *P* = 0.079), nor M class (M+ vs M0, *P* = 0.577) significantly impacted the mean adjusted patient delay.

**Table 5:** Multivariate linear model for variables predicting patient delay, primary health-care delay, specialist delay, and total delay

	Patient delay				Primary health-care delay				Specialist delay			
	Geometric mean	95% CI		*P* value	Geometric mean	95% CI		*P* value	Geometric mean	95% CI		*P* value
History of smoking												
Never smoked									60.8	47.8	77.5	0.145
Former smoker									48.9	39.8	60.1	
Current smoker									44.4	32.4	61.0	
Employment												
Currently employed or studying	34.5	10.8	110.3	0.146								
Unemployed or retired	61.9	25.0	153.2									
Site												
Anterior wall					10.3	2.8	37.8	0.143				
Lateral wall					5.6	1.6	19.4					
T Class, 7th and 8th UICC editions												
T1-T2												
T3-T4												
N class, 8th UICC edition												
N0									52.4	39.6	69.1	0.727
N+									49.6	41.8	58.8	
Stage, 7th UICC edition												
I-II	97.5	25.6	372.0	**0.009**								
III-IV	21.9	9.6	49.8									
p16 status												
Positive									53.7	43.0	67.1	0.544
Negative									48.3	37.1	62.9	
Histological grade												
I												
II												
III												
Number of different symptoms												
One	32.7	10.8	99.0	0.047*								
Two	34.0	10.7	108.1									
Three or more	88.9	31.9	247.9									
Initial place of visit												
GP, dentist, or hospital emergency					11.8	4.1	25.8	0.164				
Private otolaryngologist					4.9	1.0	24.3					
Was a follow-up visit scheduled?												
No					5.0	1.6	15.8	0.119				
Yes					11.4	2.7	49.0					
Number of health-care visits before referral to specialist care												
One					2.4	0.7	8.4	< 0.001**				
Two					10.6	2.7	42.0					
Three or more					17.0	4.1	71.0					
Appointment at another hospital												
No									47.9	40.2	57.1	0.343
Yes									54.2	42.4	69.2	
Treatment modality												
Surgery with or without (C)RT									46.6	37.2	58.4	0.141
(C)RT									55.6	46.0	67.4	
Treatment intent												
Curative	27.2	15.4	47.9	0.191	14.8	7.2	30.5	0.179				
Palliative	78.6	14.7	420.7		3.9	0.5	30.9					

Bonferroni-corrected *P* values: *, One vs three or more *P* < 0.081; **, One vs two *P* value 0.007, one vs three or more *P* < 0.002

*GP* general physician, *(C) RT* (chemo)radiotheraphy

The median PHC delay was 15 days (mean, 43; range, 0 days–2.5 years). The univariate analysis revealed that neither patient- nor tumor-related factors nor symptoms had any impact on PHC delay. Other factors that significantly correlated with PHC delay included whether a follow-up visit was scheduled, the number of visits to a doctor before referral to Dept. ORL-HNS (Table 6). According to the covariate-adjusted analysis, the number of visits to a doctor before referral to the SC clinic remained a significant predictor of a longer PHC delay.

**Table 6:** Factors influencing health care-related delay (*n* = 83 patients)

	Number (%)	PHC^a^ delay	*P* value	Specialist-care delay^b^	*P* value
Initial place of visit			0.088		
General physician, dentist or hospital emergency	71 (85.5)	16.0			
Private otolaryngologist	10 (12.0)	1.0			
Unknown	2 (2.4)				
Was a follow-up visit scheduled?			**0.012**		
No	16 (19.3)	37.5			
Yes	65 (78.3)	14.0			
Unknown	2 (2.4)				
Number of health-care visits before referral to specialist care			**< 0.001**		
One	37 (44.6)	1.0			
Two	22 (26.5)	20.0			
Three or more	16 (19.3)	33.0			
Unknown	8 (9.6)				
Appointment at other hospital					0.066
No	60 (72.3)			49.0	
Yes	23 (27.7)			63.0	
Treatment modality					0.112
Surgery with or without adjuvant (chemo-)radiation	30 (36.1)			46.0	
(Chemo-)radiation	53 (63.9)			55.0	
Treatment intent			**0.030**		
Curative	77 (92.8)	17.0			
Palliative	6 (7.2)	0.5			

^a^*PHC* primary health care

^b^*n* = 77 patients

The median SC delay was 54 days (mean, 59; range, 12–231). The median delay between referral to the SC clinic and the first appointment with an otorhinolaryngologist – head and neck surgeon was 7 days (mean, 12; range, 0–153). The median delay between the referral and diagnosis was 16 days (mean, 28; range, 0–237) and the median treatment delay was 29 days (mean, 30; range, 0–73). Treatment modality played a significant role on the delay between diagnosis and the initiation of curative treatment. Patients treated surgically, with or without adjuvant (chemo-)radiotherapy ([C]RT), had a significantly shorter unadjusted median treatment delay than patients treated with definitive (C) RT (15 days vs 37 days; *P* < 0.001). Among surgically treated patients, 90% initiated treatment within 35 days. The corresponding delay among patients treated with (C) RT reached 54 days. None of the patient- or tumor-related factors significantly impacted SC delay. Patients reporting a lump on the neck had a significantly shorter SC delay (Table 4). Yet, none of the variables significantly predicted the SC delay in the covariate-adjusted analysis.

Patients (*n* = 6; 7%) who only received palliative treatment experienced a significantly longer patient delay (119 vs 28 days; *P* = 0.023) and shorter PHC delay (17 vs 1 day; *P* = 0.030). More specific data related to delays among patients who received palliative treatment appear in Supplementary Table 7.

A patient delay of more than 6 months occurred in 9 cases, but no clear pattern of medical care–seeking behavior emerged (Supplementary Table 8). A PHC delay of more than 3 months occurred in 8 cases. In 4 of those cases, the referral to SC was delayed because of a false benign finding upon ultrasound or a fine-needle biopsy result (Supplementary Table 9).

We also reviewed patient data (*n* = 28) among those who did not complete the questionnaire. We found no difference in the age and sex distributions among patients who completed the questionnaire (*n* = 83) compared with patients who did not (*n* = 28). Patients who did not complete the questionnaire had a generally larger primary tumor (T3–4; 39.3% vs 26.5%) and more advanced stage according to UICC 8th edition (stage III–IV; 42.8% vs 20.3%). Furthermore, HPV-unassociated OPSCC was more prevalent in the no questionnaire patient group (39.3% vs 20.5%) and more patients received palliative treatment (25.0% vs 7.2%).

## Discussion

Patient, PHC, and SC all influence the length of delay between the first symptoms and the initiation of curative treatment. In our study cohort of 83 OPSCC patients, the most important factors influencing delays before treatment were the tumor stage, the number of presenting symptoms, and the number of visits to a doctor before a referral to the Dept. ORL-HNS. Interestingly, p16 status played no role on that delay, although distinctive differences between two patient groups emerged regarding patient age, smoking status, alcohol use, tumor stage and the histological grade.

In Finland, with a population of 5.5 million, the management of HNC is regulated by governmental authorities and organized by the public health care system for all patients and, more importantly, centralized at five university hospitals. Patients can either seek medical care from the public PHC centers or from private medical care centers offering SC. Both sectors, will similarly be able to refer patients to public SC. In public sector, patient’s care and the referral pattern are similar throughout the entire country and the health care is almost entirely funded by the municipality that the patient belongs to. Only a small portion is paid by the patient and the payment limit for any health care services in Finland is 683 euros during a calendar year, after which all care is entirely free for the patient. The Finnish Ministry of Social Affairs and Health in its 2010 working group report dictated that the SC delay from HNC diagnosis should not exceed 3 weeks [25]. Furthermore, some other European countries have also assessed guidelines for the timely initiation of HNC treatment. Our findings offer information which can further minimize any SC delays. Both increased patient awareness and expedited health care processes warrant continuous developmental efforts.

Most patients had HPV-associated OPSCC (79%) and diagnostic time intervals did not differ according to the p16 status. These findings are in line with a recent North American retrospective cohort study of 152 OPSCC patients, where 84% of the tumors were HPV positive. In that study, the median patient and PHC delays were 21 days and 8 days, respectively [26]. According to a recent meta-analysis regarding patients with oral cancer, the weighted mean patient delay was 80.3 days [27], which is significantly longer than in our study (median, 30 days; mean, 56 days). In many studies, the delay data are shown as means, even though patient delay distribution skews to the right and abruptly chosen cut-off points are used to divide patients into two or three delay groups for analysis, which can bias the findings [28–30]. For these reasons, we present our results as medians and analyzed delay data as continuous variables.

Symptoms that significantly correlated with a prolonged patient delay in univariate analysis consisted of weight loss and difficulties breathing. It might seem counterintuitive that such a severe symptom as breathing difficulty does not force patients to seek medical care immediately. However, in our previous study [22], we reported a similar finding, whereby patients’ interpretations of breathing difficulty varied. If the patient reported more than one symptom, the sequence and time of symptom emergence remained unknown. Patients might have experienced milder symptoms initially, and, therefore, postponed seeking medical care.

Patients with a more advanced disease at the time of diagnosis experienced a shorter patient delay. The heterogeneity of malignancies and their varying biological behavior cause differences in their clinical course. Cancers that metastasize rapidly to regional lymph nodes might cause more notable symptoms and, therefore, may lead to a shorter patient delay. Slowly growing tumors without regional lymph node involvement might lead to a slower progression and the emergence of symptoms and, thus, lead to a longer delay. This naturally remains highly speculative, such that our results indicated that a lump on the neck only shortened the SC delay. However, among TNM variables, N class emerged as the most strongly associated with patient delay in a covariate-adjusted analysis, implying that a lump on the neck might cause a patient to seek medical care promptly. Yet, this correlation remained insignificant in our analyses. Existing literature provides varying results regarding the association between cancer stage and patient delay [12, 14, 29–32]. Multiple studies on head and neck tumors show no correlation between patient delay and tumor size [12, 32, 33]. We chose to use both the UICC 7th and 8th editions in our analysis, because the new edition was released in the middle of our prospective data collection, and we wanted to be able to compare our results with the existing literature and review our data according to the newest classification. We can assume that the UICC 7th edition for tumor classification better represents the clinical findings of disease, because the N classification varies according to the number and size of metastatic lymph nodes on the ipsilateral side of the tumor regardless of the HPV status. The UICC 8th edition focuses more on disease prognosis. In this regard, the UICC 7th edition better associates with factors and clinical findings influencing patients’ medical care–seeking behavior, allowing for comparison between the clinical findings among the two patient groups.

HPV-associated OPSCC carries a better overall and progression-free survival than HPV-unassociated OPSCC [3, 7–10]. Patients with HPV-associated OPSCC tend to have a smaller primary tumor, but more extensive neck disease [7–9]. A recent study on patients’ initial symptoms stated that two-thirds of patients with HPV-associated OPSCC presented with an asymptomatic neck mass as the initial symptom [34]. Another study showed that among patients with HPV-unassociated OPSCC, symptoms were more often related to the primary tumor site, including a sore throat and dysphagia or odynophagia [35]. Based on these findings, it seems logical that HPV association would also impact delays. The baseline characteristics in our patient cohort agree with those presented in previous studies, since p16-positive patients were generally younger, smoked fewer cigarettes, and consumed less alcohol [6, 7]. Remarkably, in a recent study by Carpén et al. [36], smoking was rather common among patients with HPV-associated OPSCC, since 63% of such patients smoked. Rather notably, 77% of the patients in our study had HPV-associated OPSCC, a markedly higher portion than about a decade ago at our institution [37], a change that mirrors observations from other Western countries.

Currently, employed or student patients reported a shorter unadjusted median patient delay compared to unemployed and retired patients. Other sociodemographic factors (i.e., sex, age, place of residence) played no role on patient delay, which agrees with other recent studies [12, 14, 33, 38, 39]. In Finland, employed individuals often have access to occupational health care in addition to the national public health care system, which might explain such a difference. However, in a multivariate analysis, this finding remained insignificant.

The most important factor that emerged affecting PHC delay involved whether a follow-up visit was scheduled during the initial visit to PHC, a finding similar to our previous study [22]. The correlation between the number of health care visits and the PHC delay is self-explanatory, since it takes time to visit a doctor during multiple appointments. Because the questionnaire was administrated after the cancer diagnosis, a possibility for recall bias exists. In order to minimize it, we double-checked the delay data from hospital records. We found that the reason for four of the five longest PHC delays consisted of a false-benign finding on a cytological or histopathological biopsy. In order to minimize the longest PHC delays, more education is needed among PHC doctors to acknowledge the risk of false-benign findings during such examinations.

Patients receiving palliative treatment typically present with more extensive disease and more notable symptoms. Therefore, we somewhat expected their PHC delays would remain fairly short. The correlation between longer patient delays among palliative care patients is more complex, since a longer delay may lead to tumor growth and disease advancement [12, 19, 40]. But, other reasons might guide management towards palliative care, such as a poor overall health, the heavy use of alcohol or an unwillingness to commit to the recommended treatment.

SC delay is multifactorial. After referral to Dept. ORL-HNS, the patient visits an otorhinolaryngologist – head and neck surgeon, who performs a clinical exam and evaluates the need for additional imaging and biopsies. Because of the multiple steps needed to ensure the best possible care, some delay is unavoidable. The median delay from the referral to SC to the initiation of curative treatment reached 50 days. This delay can be divided into the time from referral to diagnosis, which amounted to approximately one-third of that delay and the time from diagnosis to treatment, which comprised two-thirds of the total delay. The median treatment delay of 29 days (15 days for surgically treated patients and 37 days for those treated with (C)RT) was similar to a large American study consisting of 51,655 HNC patients in which the median treatment delay for patients treated with surgery, RT and CRT were 17, 31 and 34 days, respectively [15]. At our institution, patients treated surgically received treatment significantly faster than patients treated with definitive (C)RT. This additional delay is partially attributed to all preparatory actions required before (C) RT, although institutional treatment resources might play a role. Nevertheless, the median delay of 35 days from diagnosis to the initiation of definitive (C) RT calls for monitoring functions and further evaluation. These actions remain important to minimizing any delay in OPSCC management.

### Limitations

We acknowledge that our patient cohort remained limited because of the low incidence of OPSCC, but we gathered data from all newly diagnosed OPSCC patients treated at our tertiary care hospital covering a catchment area with a population of 1.6 million people (almost one third of the whole country) and 75% of these patients were able and willing to complete the administrated questionnaire. The data from the remaining patients were gathered from the hospital records. Our study setting allowed for structured and comprehensive data collection. Patients not included in this study had a generally more advanced disease and palliative treatment was more prevalent among these patients, possibly resulting in some selection bias reflected in the findings. We excluded patients unable to complete the questionnaire or who did not return it from the analysis, which is an unavoidable limitation to this kind of study. The questionnaire was administrated following cancer diagnosis and, therefore, may have affected patients’ abilities to correctly recall symptom onset.

## Conclusions

In total, the patient and management delay for OPSCC was shorter than expected since most patients were promptly referred to SC. We found that p16 status did not affect the delay. The most important factors influencing treatment delays included tumor stage, the number of different symptoms, and the number of visits to a doctor before the patient was referred to SC. The treatment delay was significantly longer for patients treated with definitive (C) RT than for those treated surgically with or without adjuvant (C)RT. However, the delay in SC remains multifactorial and requires monitoring by an institutional quality control system.

## Supplementary information

**Additional file 1: Table 7.** Patients who received palliative treatment

**Additional file 2: Table 8.** Patient delays exceeding 6 months

**Additional file 3: Table 9.** Primary health-care (PHC) delays exceeding 3 months

## Data Availability

The datasets generated during and/or analysed during the current study are not publicly available due to the fact that data are generated based on patient questionnaires in Finnish and individuals may be recognized based on their answers. These data are available from the corresponding author on reasonable request.

## Abbreviations

OPSCC: Oropharyngeal squamous cell carcinoma
HPV: Human papillomavirus
HNC: Head and neck cancer
PHC: Primary health care
SC: Specialist-care
Dept. ORL-HNS: Department of Otorhinolaryngology – Head and Neck Surgery
(C)RT: (Chemo-)radiotherapy
